# Correlated amplitudes of potentials evoked in homologous muscles by magnetic stimulation reveal positive covariation of corticospinal output

**DOI:** 10.1113/EP093276

**Published:** 2025-10-26

**Authors:** Richard G. Carson

**Affiliations:** ^1^ Trinity College Institute of Neuroscience and School of Psychology Trinity College Dublin Dublin Ireland

**Keywords:** brain stimulation, corpus callosum, cortex, inter‐hemispheric facilitation, inter‐hemispheric inhibition, motor control

## Abstract

It is widely held that in human, fibres of the corpus callosum mediate inter‐hemispheric inhibition – deemed necessary to prevent a bilateral cerebrum from generating simultaneous and potentially conflicting outputs. Ostensible support comes from an electrophysiological phenomenon whereby the mean magnitude of ‘test’ motor evoked potentials (MEPs) obtained in response to magnetic stimuli delivered over the contralateral motor cortex is diminished when initial ‘conditioning’ magnetic stimuli have been applied 6–15 ms previously to the opposite motor cortex. A contrary view is that this phenomenon masks, rather than reveals, normal physiological processes. An alternative hypothesis is that cortical motor centres giving rise to efferent projections onto motoneurons innervating homologous muscles conduct reciprocal shaping of excitation. This hypothesis was examined in a large sample (205 participants) by correlating the amplitude of MEPs elicited by a conditioning stimulus (CS) with the amplitude of those elicited 10 ms later by a test stimulus (TS). The magnitudes of responses to the CS and TS were positively correlated. This remained the case following statistical compensation for an observed covariation of low amplitude fluctuations in the background (<2 µV root mean squared) electromyographic activity recorded in the (homologous) target muscles prior to stimulation. Although the coefficients representing the magnitude of association between responses to the CS and TS are small (rho < 0.20), they are reliable. These findings support the hypothesis that there is positive covariation in the excitability of corticospinal projections from the two cerebral hemispheres to homologous muscles of the upper limb.

## Abstract (highlights)

- **What is the central question of this study?**
  Is there covariation in the excitability of corticospinal projections from the two cerebral hemispheres to homologous muscles of the upper limb?
- **What is the main finding and its importance?**
  There is positive covariation in the amplitudes of motor evoked potentials generated by near simultaneous stimulation of the two motor cortices, some of which arises from correlated bilateral fluctuations in the excitability of spinal motoneurones.

## Graphical abstract

**Abstract figure** There is positive covariation in the amplitudes of motor evoked potentials (MEPs) generated by (near simultaneous) stimulation of the two motor cortices. That is, larger responses to stimulation of the left motor cortex tend to be accompanied by larger responses to stimulation of the right motor cortex, and smaller responses to stimulation of the left motor cortex tend to be accompanied by smaller responses to stimulation of the right motor cortex. Part of this covariation arises from correlated bilateral fluctuations in the excitability of spinal motoneurones. It nonetheless remains consistently expressed following statistical compensation for these fluctuations. The presence of positive covariation does not accord with the conjecture that each cerebral hemisphere has a suppressing influence upon its opposite counterpart. This artwork is available at: https://commons.wikimedia.org/wiki/File:Covariation_in_the_excitability_of_corticospinal_projections.jpg. It contains elements derived from the following source: https://scidraw.io/drawing/754 (https://zenodo.org/records/14219075). These elements were published under a Creative Commons Attribution License (CC BY).

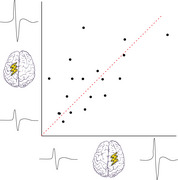

## 1 INTRODUCTION

There is a widespread belief that, in human, each cerebral hemisphere has a suppressing influence upon its opposite counterpart. To this conjecture, the term ‘interhemispheric inhibition’ has been applied (e.g. Chiarello & Maxfield, 1996). An associated presumption is that its function is to prevent a bilateral cerebrum from generating simultaneous and potentially conflicting outputs (e.g. Hellige, 1993; Moscovitch, 1976). Although the development of theoretical models that accord a central role to this hypothesized process (e.g. Kinsbourne, 1974) preceded the innovation of transcranial magnetic stimulation (TMS) (Barker et al., 1985), studies utilizing this technology are frequently invoked in the guise of corroborating evidence (e.g. Duque et al., 2007). The TMS variant that usually serves this role takes the form of an initial ‘conditioning’ stimulus (CS) applied over the cortical motor area of one hemisphere, followed 6–15 ms later by the delivery of a ‘test’ stimulus (TS) over the matching site on the opposite side of the head. Both the CS and the TS are delivered at intensities that, when used in isolation, are sufficient to induce motor evoked potentials (MEPs) in muscles of the contralateral limb. The prototypical finding (Ferbert et al., 1992) is that the average magnitude of MEPs produced by a series of responses to the TS is reduced if in each case there is a preceding CS. For this very specific electrophysiological phenomenon, the term ‘inter‐hemispheric inhibition’ (IHI) has also been adopted. This is unfortunate, as in the mind of the unwary, the similitude of terminology may create the impression that the two phenomena (one theoretical, the other empirical) are the same. A more insidious risk is that a diminution of average MEP amplitude brought about by a specific ‘paired‐pulse’ TMS methodology is interpreted as support for the conjecture that the cerebral hemispheres instantiate reciprocal suppression.

It has been argued previously (Carson, 2020) that in an IHI protocol, the intense transsynaptic bombardment of neurons in the opposite hemisphere to which the CS gives rise promotes a massed and undifferentiated instantiation of inhibitory processes, unlike that which occurs in normal physiological conditions (see also Bäumer et al., 2006; Ferbert et al., 1992; Hanajima et al., 2001). When assessed over the course of several trials, the net effect of this barrage is quantifiable as the decrease in the average magnitude of MEPs elicited by the TS, relative to that calculated for trials without a CS. As noted by Asanuma and Okamoto (1959) in their detailed studies in cat, should a relatively large number of callosal fibres be activated (i.e. by artificial means), a focal excitatory effect may be masked by the overlapping and summative influence of fibres that project onto interneurons in the opposite hemisphere, which mediate the expression of local (surround) inhibition.

There are, however, indications that in some very specific circumstances, focal excitatory inter‐hemispheric effects can be revealed in human by means of TMS. In a protocol otherwise resembling that used to obtain IHI, a subthreshold CS (i.e. below the intensity required to evoke a volley that descends to the spinal cord) precedes the TS by 3–8 ms (Ferbert et al., 1992; Hanajima et al., 2001; Ugawa et al., 1993). Using certain stimulation conditions (e.g. Bäumer et al., 2006), a net increase in the average amplitude of MEPs elicited by the (suprathreshold) TS is obtained (i.e. expressed relative to the average obtained in response to the TS alone). Employing an innovative analysis of data acquired using a conventional IHI protocol, Belyk et al. (2021) estimated the magnitude of inhibition as the difference between the amplitude of each conditioned MEP and the median amplitude of the MEPs obtained for all trials on which there was no CS. An inverse relationship was observed to exist between this (per trial) estimate of IHI and the amplitude of the MEP generated by the CS. That is, on trials on which the response to the CS was larger, the magnitude of the inhibition tended to be smaller. The authors referred to this as ‘paradoxical facilitation’.

It might be argued that this effect appears paradoxical only if the phenomenon of IHI is taken to reflect normal physiological interactions between the cerebral hemispheres. An alternative view is that the (‘paradoxical’) effect reflects the presence of homotopic excitatory transcallosal projections. It has been proposed that these projections form part of a functional unit that comprises a ‘facilitatory centre and a depressing periphery’ (Bianki, 1981) that (in the target hemisphere) sculpts the influence of converging inputs to pyramidal neurons (Carson, 2020). In this scheme, since reciprocal shaping of excitation in brain centres (i.e. in both cerebral hemispheres) with connections (e.g. via the corticospinal tract) onto motoneurons innervating homologous muscles is assumed, positive covariation in the amplitudes of MEPs generated by the near simultaneous stimulation of the two motor cortices would be predicted. The first aim of the present study was to evaluate this prediction and, using a very large sample, obtain precise estimates of the extent of any covariation that might be present.

Since the initial pioneering studies of motor cortex stimulation in human (e.g. Rothwell et al., 1987), it has been appreciated that even subtle variations in background muscle activity have dramatic effects on the magnitude of the potential that is evoked. That the excitability of the spinal motoneurones plays a determining role, is further emphasized by similarities in the extent to which responses to transcranial electrical (Hess et al., 1987) cervicomedullary (Maertens de Noordhout et al., 1992) and cortical magnetic stimulation are potentiated by weak (≤5% maximum) voluntary contractions. As emphasized by Matthews (1999, p. 868) ‘the cortical response is normally viewed via its actions on motoneurones and their contribution has to be assessed before understanding can be achieved’. It is possible to demonstrate bilateral synchronization of EMG recorded at rest from homologous upper (Kavanagh et al., 2013) and lower (Boonstra et al., 2008) limb muscles. Does covariation in the amplitudes of MEPs generated by stimulation of the two motor cortices arise from correlated bilateral fluctuations in the excitability of spinal motoneurones? The second aim of the present study was to address this question.

## 2 METHODS

### 2.1 Participants

Two hundred and five neurologically healthy volunteers (aged 18–26 years, mean age = 21.8 years, standard deviation = 1.37 years; 117 females) were engaged. None reported a history of neurological/psychiatric disease, or the use of any medication considered to be a contraindication for TMS (Rossi et al., 2021). Based on responses to the Edinburgh Handedness Inventory (Oldfield, 1971) all were deemed to be right‐handed. Participants provided written informed consent to the procedures, which were approved by the Trinity College Dublin School of Psychology Research Ethics Committee (SPREC102011‐01). Excepting study pre‐registration, all testing was undertaken in accordance with the Declaration of Helsinki. The procedures described in the present report were conducted before the start of a motor learning protocol. The duration of the entire session was approximately 3 h. The participants returned 1 week later for an additional session of approximately 30 min duration. In recognition of this commitment of time, each participant was given a shopping voucher to the value of 20 Euros as a token of appreciation.

### 2.2 Recording procedures

The participants were seated with their upper limbs supported. The elbows were semi‐flexed (100–120°). The forearms were placed in mid‐pronation and stabilized by vacuum cushions (Vac‐Pac Size 11, Olympic Medical, Seattle, WA, USA). The hands were secured at mid palm in custom built manipulanda. As these did not permit extension of the wrist, and flexion of the wrist was opposed by a stiffness load (∼0.67 N m/θ (rad)), the hands were effectively immobile. An orthopaedic neck brace (RC0007M, Cervical Collar, Aspen Medical Products, Irvine, CA, USA) was worn to restrict the range of head motion. Electromyographic (EMG) activity was recorded from left and right flexor carpi radialis (FCR) and extensor carpi radialis (ECR) using pairs of Ag–AgCl electrodes (Cleartrace 1700, ConMed, Haverhill, MA, USA). The electrode locations were referenced to anatomical landmarks following the procedures outlined by Perotto (1994). For the present purpose, only data recorded from the FCR muscles are reported. As surface electrodes were used, it is possible that action potentials originating in muscles other than FCR were also recorded.

A reference electrode was placed on the lateral epicondyle of the right humerus. The skin overlaying the electrode sites was prepared with a topical abrasive (Nuprep, Weaver & Co., Aurora, CO, USA) and then sanitized with an antiseptic (70% isopropanol). EMG signals were amplified (gain = 1000) by BioPac EMG100C amplifiers (Biopac Systems UK, Pershore, UK), band‐pass (100–1000 Hz) filtered (NL125/6, Digitimer Ltd, Welwyn Garden City, UK), digitized (16 bit precision; ±5 V input range) at a sampling rate of 5 kHz (Micro1401, CED, Cambridge, UK), and recorded with Signal software (Signal 7.01, CED). This permitted the analog signals to be digitized at approximately 0.15 µV resolution. The lower limit of the band pass filter was selected to eliminate movement artefacts associated with ballistic movements (e.g. Ruddy et al., 2016) performed by the participants following these recordings. It has been demonstrated (Sebik et al., 2013) that when using comparable filter settings, the electrical activity of the muscle can be resolved via surface EMG with sufficient fidelity to allow multi motor unit action potentials to be characterized. It is therefore unlikely that the band pass filter used in the present study adversely affected resolution of the features to be extracted from the EMG signals (Karacan et al., 2023).

### 2.3 Stimulation procedures

**FIGURE 1:**
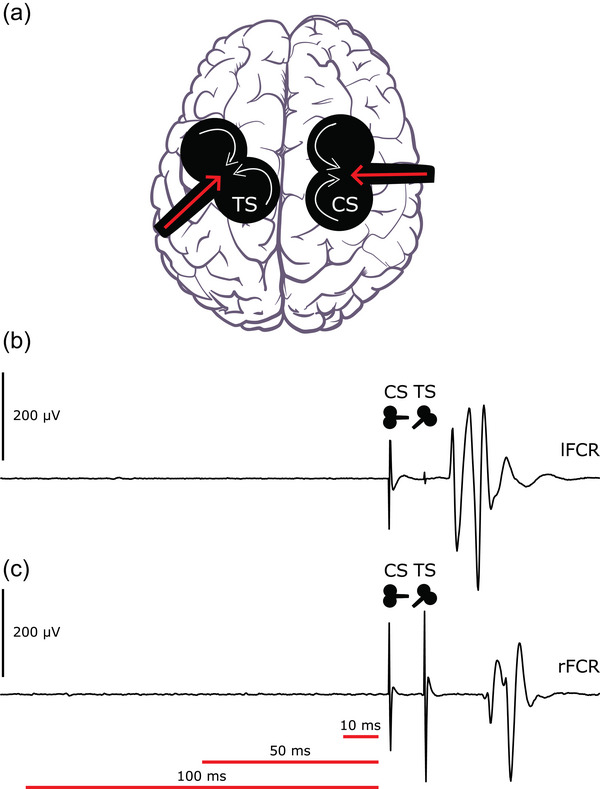
Schematic representation of the configuration used for transcranial magnetic stimulation (TMS), with exemplary responses. (a) Superior view of the brain illustrating the arrangement for dual‐site TMS. The white arrows on the TMS coils illustrate the direction in which current flowed through the coil. The red arrows indicate the direction of the induced eddy current in the cortex. The coil used to deliver the conditioning stimulus (CS) induced current that flowed in a lateromedial direction. The coil used to deliver the test stimulus (TS) induced current that flowed in a posteroanterior direction. (b) Example of a response to the CS recorded from the left flexor carpi radialis (FCR) muscle. (c) Example of a response to the TS recorded from the right FCR muscle. In (b, c), the CS annotated coil silhouette marks the artefact arising from the CS. The TS annotated coil silhouette marks the artefact arising from the TS. The red horizontal lines at the bottom of (c) indicate the three separate intervals over which the magnitude of the root mean squared electromyographic signal was calculated. In each case, the interval ended 3 ms prior to the delivery of the CS. In (b, c), a vertical scale (labelled ‘200 µV’) is provided. This artwork is available at: https://commons.wikimedia.org/wiki/File:Dual_hemisphere_TMS.jpg, It contains elements derived from the following source: https://scidraw.io/drawing/754 (https://zenodo.org/records/14219075). These elements were published under a Creative Commons Attribution License (CC BY).

Magnetic stimuli were delivered by two Magstim 200 (monophasic waveform) stimulators (Magstim Company, Whitland, UK) using figure of eight coils (internal wing diameter 70 mm). In all cases the coil used to deliver the TS was placed over the left hemisphere with the axis of intersection between the two loops oriented at approximately 45° to the sagittal plane, to induce posterior to anterior (P‐A) current flow across the motor strip (Kammer et al., 2001). The coil used to deliver the CS was placed over the right hemisphere. Consistent with IHI protocols used previously in conjunction with EMG recordings from forearm muscles (e.g. Ibey et al., 2015), this coil was oriented perpendicular to the mid‐sagittal plane, thereby inducing a lateral to medial (L‐M) directed current in the brain (Figure 1).

To determine the optimal site for stimulation (separately for each hemisphere), the output of the stimulator was increased incrementally from 30% of maximum stimulator output (% MSO) with the centre of the coil first positioned over a craniometrically defined location 2 cm anterior and 6 cm lateral to the vertex, until a MEP was visible on an oscilloscope displaying the EMG signal at high gain. Subsequently, the coil was moved in small (∼0.5 cm) increments from this position, along the anterior‐to‐posterior and mediolateral axes, and the stimulation intensity increased or reduced. The search culminated at the coil location which elicited a MEP at the lowest stimulation intensity. This location was registered by marking directly on the scalp the outline of the coil and a vector indicating the long axis of its handle. These markings were used subsequently to reproduce the position and orientation of the coil. The coil was then removed, replaced at the marked location, and stimulation applied again to verify that MEPs could still be evoked at the same intensity. If MEPs were not reliably elicited, the procedure for determining the optimal site was repeated.

During the test procedures, the stimulating coils and cables were supported either by an overhead suspension system or by articulated mechanical arms. One experimenter standing (on a pedestal) above and behind the participant held the TS coil over the left hemisphere. A second experimenter standing (also on a pedestal) to the right of the seated participant held the CS coil over the right hemisphere. Each experimenter monitored the position and orientation of the coil continuously and, if necessary, made minor adjustments to ensure that it remained in alignment with the scalp markings.

Resting motor thresholds (rMT) are typically defined as the minimum stimulation intensity required to elicit a MEP with a peak‐to‐peak amplitude of 50 µV in the target muscle (Rossi et al., 2009). For most of the participants in the present study, these were obtained using a maximum likelihood protocol – referred to as the parameter estimation by sequential testing (PEST) method, described by Awiszus (2003). This protocol utilizes a sigmoid‐shaped logistic function to determine the stimulation intensity at which there exists a 0.5 probability of eliciting a MEP with the defined peak‐to‐peak amplitude (i.e. 50 µV). The method was implemented using the MTAT 2.0 program (Awiszus & Borckardt, 2011). For fifty‐two participants, the rMT was obtained as the lowest stimulation intensity at which MEPs with peak‐to‐peak amplitude of approximately 50 µV were evoked in at least 5 of 10 consecutive trials (i.e. following Rossi et al., 2009).

### 2.4 Test protocol

For all participants included in this report, a conventional sIHI test protocol was employed. This consisted of (median = 18, minimum = 17; maximum = 20) trials during which a CS was delivered over the right hemisphere at an intensity of 130% rMT, 10 ms in advance of a TS delivered over the left hemisphere at an intensity of 120% rMT. The recording of each trial commenced 0.5 s before the TS and concluded 0.5 s following delivery of the TS. On other trials, either the response to only the CS (*n* ≥ 10 trials) or to only the TS (median *n* = 17 trials) was registered. In all cases, a second ‘paired‐pulse’ TMS protocol was included in each block of trials, that is, addition to sIHI. For 36 participants this was long IHI (lIHI); for 48 participants it was ‘inter‐hemispheric facilitation’ (IHF); for 33 participants ‘long interval intracortical inhibition’ (LICI); and for 41 participants ‘short interval intracortical inhibition’ (SICI). For a further 47 individuals, sIHI, lIHI and IHF protocols were included. The order of delivery of trials in the various conditions (e.g. sIHI, lIHI, TS only, CS only) was randomized. The interval between successive trials was approximately 8 s – ‘jittered’ by 50%, such that the range was between 6 and 10 s. The total duration of each block of (maximum = 68) trials was always less than 10 min. These data have not been published previously.

### 2.5 Data reduction

To quantify the short‐latency component of the response to the TS and the CS (e.g. Rothwell et al., 1991), the amplitude of each MEP elicited in the target muscle (respectively rFCR and lFCR) was obtained as the absolute difference between the maximum and minimum values of the EMG time series in an interval commencing 12.5 ms and concluding 45 ms following delivery of the magnetic stimulus. It should be noted that with respect to the response to the CS, the interval in which the maximum and minimum values were obtained commenced 2.5 ms following delivery of the TS. It was verified by visual inspection of every trial that any electromagnetic artefacts evident in the lFCR EMG recording, arising from the TS (delivered ipsilateral to the lFCR), had dissipated prior to the start of this interval.

The root mean square (r.m.s.) of the background EMG recorded in the rFCR and lFCR was calculated separately for three periods. The first period (100 ms duration) commenced 103 ms and ended 3 ms prior to the CS (i.e. 13 ms prior to the TS). The second period (50 ms duration) commenced 53 ms and ended 3 ms prior to the CS (i.e. 13 ms prior to the TS). The third period (10 ms duration) commenced 13 ms and ended 3 ms prior to the CS (i.e. 13 ms prior to the TS). The objective in estimating background EMG is generally to assay the post‐synaptic state of the spinal motoneurons in the closest feasible temporal proximity to the delivery of the cortical stimulus. Since data are sampled at a fixed frequency, there is necessarily a trade‐off between the limit of this proximity and the number of samples available for estimation of central tendency. Although the most (statistically) reliable estimates will be obtained for longer pre‐stimulus intervals, necessarily the initial samples will be taken further in advance of the stimulus than would be the case if a short interval was used instead. As estimates of ‘background’ EMG were integral to the data processing methods used herein, the three epochs of differing duration were used, with a view to determining whether this factor had a material bearing on the estimated covariation in the amplitudes of MEPs.

All statistical analyses were implemented in R (R Core Team, 2023).

## 3 RESULTS

It is well known that the distributions of MEP amplitude values tend to exhibit substantial deviations from normality (Nielsen, 1996). This characteristic extends to samples comprising r.m.s. EMG records derived from separate trials, particularly in circumstances (e.g. when ‘at rest’) in which a relatively small number of motor units (i.e. representing a limited portion of the motoneuron pool) are represented in the EMG signal (Rupasov et al., 2012). Indeed, skewed distributions are to be anticipated in such cases, since the sampled values are small (relative to the potential range), variances are large (reflecting the causal influence of multiple independent random processes with arbitrary statistical properties) and the values cannot be negative (Limbert et al., 2001). Given these considerations, the use of non‐parametric measures of association, such as Spearman's rho, is indicated.

As a first step in establishing whether there exists a positive association between the amplitudes of potentials evoked in homologous muscles by cortical magnetic stimulation delivered (near‐simultaneously) over the two cerebral hemispheres, the Spearman correlation method was therefore applied. For each participant a rho value was derived by correlating the pairs (median *n* = 18) of MEP amplitude values obtained for each block of trials. One element of each pair indicated the magnitude of the EMG response evoked by the TS. The other element indicated the magnitude of the response to the CS. As all pairs of values included in the correlation were obtained successively from the same participant, the presence of serial correlation (autocorrelation) cannot be excluded. It is, however, the case that while the estimated variance associated with the calculation of the coefficient may be biased by serial correlation, the coefficient estimate itself (i.e. the rho value) will be unbiased. As hypothesis testing was not undertaken at the level of each correlation coefficient, the presence of serial correlation has no bearing on the interpretation of the outcomes that are reported.

**FIGURE 2:**
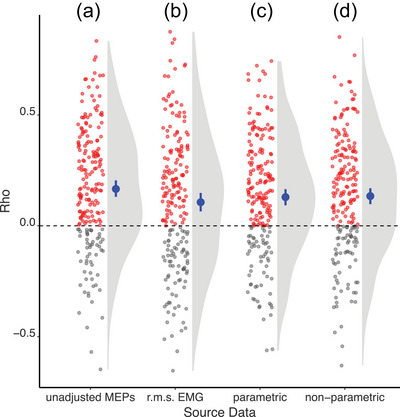
Estimates (*n* = 205 participants) of the Spearman rank correlation coefficient (rho) quantifying the degree of monotonic association between measures derived from EMG recordings of the right (rFCR) and left (lFCR) flexor carpi radialis muscles. (a) The measures correlated were the amplitude of motor evoked potentials (MEPs) elicited in the rFCR in response to a test stimulus (TS) applied over the left hemisphere, and the amplitude of MEPs elicited in the lFCR in response to a preceding conditioning stimulus (CS) applied over the right hemisphere. (b) The measures correlated were the root mean squared electromyographic signal (r.m.s. EMG) recorded in the rFCR over a 100 ms interval that ended 3 ms prior to the delivery of the CS, and the r.m.s. EMG response recorded in the lFCR over the same interval. (c) The amplitudes of the MEPs elicited in the rFCR and lFCR were entered in parametric semi‐partial correlations (see text for details) whereby the respective covariates were: (i) the r.m.s. EMG recorded in rFCR over the period 100 ms prior to the CS; and (ii) the r.m.s. EMG recorded in lFCR over the same interval. (d) The amplitudes of the MEPs elicited in the rFCR and lFCR were entered in non‐parametric semi‐partial correlations (see text for details) whereby the respective covariates were: (i) the r.m.s. EMG recorded in rFCR over the period 100 ms prior to the CS; and (ii) the r.m.s. EMG recorded in lFCR over the same interval. In all cases, positive values of the correlation coefficient are shown as red symbols. Negative values of the correlation coefficient are shown as grey symbols. The blue symbols indicate the mean values of rho (*n* = 205). The accompanying error bars represent the bootstrapped 95% confidence intervals. A kernel density estimate (derived using a Gaussian kernel) – which provides a non‐parametric model of the distribution of points, is also shown (grey shaded area) for each sample.

As inspection of Figure 2a reveals, evaluated across the sample of 205 participants, there exists a positive monotonic association between the amplitudes of MEPs generated on the same trial by the TS and the CS. That is, larger responses to the CS tended to be accompanied by larger responses to the TS. And smaller responses to CS tended to be accompanied by smaller responses to the TS. Binomial expansions (Ferguson, 1971) indicate that the probability of obtaining through chance alone the observed balance of positive and negative correlation coefficients is extraordinarily low (7.29 × 10^−12^, Table 1). The point estimate of rho (i.e. the mean of all participants), in conjunction with the corresponding 95% confidence interval (Table 1), similarly suggests that the presence of a positive association in the population can be inferred with a relatively high degree of certainty.

**TABLE 1:** Spearman rank correlation coefficient (rho) for the association between the amplitude of MEPs elicited in lFCR and rFCR.

		95% CI					
	Mean rho	Lower	Upper	+ve (*n*)	−ve (*n*)	*p* (binomial)	BF_10_
Unadjusted	0.17	0.13	0.20	150	55	7.29 × 10^−12^	
Parametric semi‐partial							
Adjusted (100 ms period)	0.13	0.09	0.17	145	60	7.89 × 10^−10^	5611
Adjusted (50 ms period)	0.15	0.11	0.19	144^*^	59^*^	6.57 × 10^−10^	25
Adjusted (10 ms period)	0.14	0.10	0.18	142^*^	62^*^	6.08 × 10^−9^	33
Non‐parametric semi‐partial							
Adjusted (100 ms period)	0.13	0.10	0.17	137	68	4.25 × 10^−7^	20175
Adjusted (50 ms period)	0.14	0.11	0.18	138	67	2.10 × 10^−7^	81
Adjusted (10 ms period)	0.14	0.10	0.18	142^*^	62^*^	6.08 × 10^−9^	277

For each participant, a Spearman rank correlation coefficient (rho) was calculated to quantify the degree of monotonic association between the amplitude of motor evoked potentials (MEPs) elicited in the right flexor carpi radialis (rFCR) muscle in response to the test stimulus (TS) and the amplitude of MEPs elicited in the left FCR (lFCR) in response to the conditioning stimulus (CS). Across all (*n* = 205) participants, the median number of trials included in each correlation was 18. The (bootstrapped) means of the rho values obtained from all participants are shown, along with the associated 95% lower and upper confidence limits. The number of positive and negative rho values generated are indicated by +ve (*n*) and −ve (*n*). respectively. The asterisks denote cases in which at least one participant had a rho value equal to zero. The probability of obtaining by chance alone the sample distribution of positive and negative coefficients is shown as *p* (binomial). The values are given first for the correlations derived without the inclusion of covariates (Unadjusted). Semi‐partial corelations were also calculated, whereby the root mean squared (r.m.s.) EMG recorded in rFCR over a period (100, 50 or 10 ms) in advance of the magnetic stimulation was included as a covariate for the rFCR MEP amplitude, and the r.m.s. EMG recorded in the lFCR over the same period was included as a covariate for the lFCR MEP amplitude. The adjustment for covariates was undertaken using both parametric and non‐parametric methods (see text for details). The B_10_ column gives the Bayes factors for sign tests that contrasted the unadjusted rho value generated for a participant, with the corresponding (adjusted) rho value derived for each instantiation of a semi‐partial correlation. All sign tests included data from 205 participants.

It was also the case that positive correlations between the r.m.s. EMG recorded in the right FCR and that recorded in the left FCR occurred more frequently than negative correlations. The magnitudes of the correlations obtained when the r.m.s. EMG values were calculated for a 100 ms period prior to the (conditioning) stimulus are shown in Figure 2b. Although the point estimate of rho is small (0.11), there is a 95% probability that the confidence interval (0.06, 0.15) contains the true value of correlation coefficient in the population (Table 2). Binomial expansions similarly indicate a low probability that the observed sample distribution of positive and negative correlations was obtained by chance. Positive monotonic associations were also more frequent than negative associations when the r.m.s. EMG was calculated for periods of 50 and 10 ms prior to the delivery of TMS (Table 2). Summaries of the r.m.s. EMG values from which the measures of association were derived are shown in Tables 3 and 4. The values in Table 3 are the means (across 205 participants) of the median r.m.s. values obtained for each participant across all trials (typically *n* = 18). The values in Table 4 are the means (across 205 participants) of the maximum r.m.s. values obtained for each participant across all trials. For most participants, the median values were less than 1µV and the maximum values less than 2 µV. Both are well below the screening thresholds that are applied commonly to exclude trials on which ‘background EMG’ is present. These values are, however, comparable to those obtained previously for the FCR muscle, with the hands and forearms stabilized effectively, when different amplification systems and filter setting have been used by our research team (e.g. Calvert & Carson, 2023) and in other laboratories (e.g. Vercauteren et al., 2008; Zult et al., 2015). Evidently, covariation of the amplitude of EMG signals recorded (‘at rest’) from homologous muscles of the upper limb can be detected when the range over which the signal amplitudes extend is less than 2 µV. Beyond the novelty of this finding, there is a practical consideration pertinent to the present investigation. The presence of positive correlations between the r.m.s. EMG recorded from the muscles in which the motor potentials were evoked prevents a determination that the amplitudes of responses to cortical stimulation are correlated only by virtue of supraspinal processes. The associations may be attributable, at least in part, to covarying changes in the post‐synaptic state of the innervating spinal motoneurons.

**TABLE 2:** Spearman rank correlation coefficient (rho) for the association between the r.m.s. EMG recorded in lFCR and rFCR.

		95% CI				
Period	Mean rho	Lower	Upper	+ve (*n*)	−ve (*n*)	*p* (binomial)
100 ms	0.11	0.06	0.15	129	76	5.64 × 10^−5^
50 ms	0.10	0.06	0.13	134	71	3.12 × 10^−6^
10 ms	0.06	0.02	0.09	115	90	0.012

For each participant, a Spearman rank correlation coefficient (rho) was calculated to quantify the degree of monotonic association between the root mean squared (r.m.s.) EMG recorded in the right flexor carpi radialis (rFCR) muscle over a period (100 ms, 50 ms or 10 ms) in advance of the conditioning stimulus and the r.m.s. EMG recorded in the left FCR (lFCR) over the same period. Across all (*n* = 205) participants, the median number of trials included in each correlation was 18. The (bootstrapped) means of the rho values obtained from all participants are shown, along with the associated 95% lower and upper confidence limits. The number of positive and negative rho values generated are indicated by +ve (*n*) and −ve (*n*). respectively. The probability of obtaining by chance alone the sample distribution of positive and negative coefficients is shown as *p* (binomial).

**TABLE 3:** Means (across 205 participants) of the median r.m.s. values obtained for each participant across all trials.

		95% CI	
Target muscle	Mean r.m.s. (µV)	Lower limit	Upper limit
Period = 100 ms			
Right FCR	0.72	0.67	0.81
Left FCR	0.77	0.72	0.89
Period = 50 ms			
Right FCR	0.70	0.66	0.79
Left FCR	0.76	0.71	0.87
Period = 10 ms			
Right FCR	0.66	0.63	0.74
Left FCR	0.73	0.68	0.86

For each participant, a median value for the r.m.s. EMG recorded in the target FCR muscle in the specified period prior to the magnetic stimulus was derived from all available trials. Across all (*n* = 205) participants, the median number of trials included in these calculations was 18. The values shown in the table correspond to the means of these median values estimated across all participants. Lower limit and upper limit refer, respectively, to the lower and upper bounds of the 95% confidence interval for the mean. The first period (100 ms duration) commenced 103 ms and ended 3 ms prior to the CS (i.e. 13 ms prior to the TS). The second period (50 ms duration) commenced 53 ms and ended 3 ms prior to the CS (i.e. 13 ms prior to the TS). The third period (10 ms duration) commenced 13 ms and ended 3 ms prior to the CS (i.e. 13 ms prior to the TS). The values are given in units of microvolts (µV).

**TABLE 4:** Means (across 205 participants) of the maximum r.m.s. values obtained for each participant across all trials.

		95% CI	
Target muscle	Mean r.m.s. (µV)	Lower limit	Upper limit
Period = 100 ms			
Right FCR	1.52	1.33	1.86
Left FCR	1.47	1.28	1.78
Period = 50 ms			
Right FCR	1.66	1.42	2.12
Left FCR	1.55	1.34	1.89
Period = 10 ms			
Right FCR	1.63	1.42	1.94
Left FCR	1.63	1.43	1.91

For each participant, a maximum value for the r.m.s. EMG recorded in the target FCR muscle in the specified period prior to the magnetic stimulus was derived from all available trials. Across all (*n* = 205) participants, the median number of trials included in these estimates was 18. The values shown in the table correspond to the means of these maximum values calculated across all participants. Lower limit and upper limit refer, respectively, to the lower and upper bounds of the 95% confidence interval for the mean. The first period (100 ms duration) commenced 103 ms and ended 3 ms prior to the CS (i.e. 13 ms prior to the TS). The second period (50 ms duration) commenced 53 ms and ended 3 ms prior to the CS (i.e. 13 ms prior to the TS). The third period (10 ms duration) commenced 13 ms and ended 3 ms prior to the CS (i.e. 13 ms prior to the TS). The values are given in units of microvolts (µV).

It is, however, possible to use statistical modelling as a means of addressing this issue. The aim is to remove from estimates of the association between MEP amplitudes the potential influence of variations in the post‐synaptic state of the spinal motoneurons. The classical means of achieving this objective is through covariate adjustment (i.e. semi‐partial correlations). In the case of parametric methods (such as Pearson's), the conventional approach consists of two steps: (1) regress each variable on the relevant covariate(s) using a linear regression model, and (2) estimate the correlation between the residuals generated by the (two) linear regression models. For non‐parametric (e.g. rank‐based) methods such as Spearman's, the challenge lies in determining an appropriate means of estimating the residuals. Two approaches to this challenge were adopted here. In the first approach, the MEP amplitude and r.m.s. EMG estimates were first subject to a Yeo–Johnson power transformation, with the intent of normalizing the sample distributions. A standard linear regression model was then applied (separately for each participant) to regress the MEP amplitude values on the corresponding r.m.s. EMG values. The residuals derived from the linear models (for rFCR and for lFCR) were then correlated using the Spearman method. In the second approach (Liu et al., 2018), the framework established by Li & Shepherd (2012) and Shepherd et al. (2016) was used to generate a form of residual (probability‐scale residuals) tailored for ordinal regression models. In the present implementation, the MEP amplitude and r.m.s. EMG estimates were first ranked (separately for each participant). An ordinal regression model (implemented using the orm() function from the rms package; Harrell, 2015) was then used to regress the ranked MEP amplitude values on the corresponding ranked r.m.s. EMG values. The (probability‐scale) residuals were extracted from the ordinal model using the presid() function in the PResiduals package (Liu et al., 2020). The residuals obtained for the rFCR were then correlated with those derived for the lFCR, using the Spearman method. Thus, for each participant two estimates of the covariate (r.m.s. EMG) adjusted Spearman correlation coefficient were generated. For the present purposes, the first method is designated parametric, as a linear relation between the Yeo–Johnson transformed variables is assumed. The second method is entirely non‐parametric, involving no such assumptions. The patterns of outcomes yielded by the two methods differed only marginally.

As inspection of Figure 2c, d reveals, when the r.m.s. EMG values calculated for a 100 ms period prior to the (conditioning) stimulus were included as a covariate, the magnitudes of the correlation coefficients were lower than the unadjusted values shown in Figure 2a. This was also the case when the covariate was based on the EMG recordings over the 50 or 10 ms period preceding the stimulus (Table 2). To quantify the consistency with which the magnitude of the rho value was diminished by the inclusion of the covariate, Bayesian sign tests were undertaken (using the dfba_sign_test() function from the DFBA package; Chechile, 2020). The resulting Bayes factors (often written as BFB_10_) can be interpreted as an indication of the strength of evidence (the odds) in favour of the hypothesis that the covariate adjusted rho values are different from the unadjusted values, relative to the null hypothesis that the adjusted and unadjusted rho values do not differ.

It is judged to be both a strength and a weakness of the Bayesian approach that an individual can have their own view of how the odds represented by a Bayes factor might be interpreted. Guidelines have been offered with the intent that Bayes factors may be comprehended in a scientific context. Wetzels and Wagenmakers (2012) propose, for example, that Bayes factors in the range 10–30 be considered ‘strong evidence’, in the range 30–100 ‘very strong evidence’, and greater than 100 ‘decisive evidence’. Ultimately it is for the reader to decide how to interpret the odds reported in Table 1. The correlation coefficient generated by the non‐parametric method (100 ms pre‐stimulus EMG as covariate) was lower than the unadjusted coefficient in 128 participants (and higher in 73 participants, with 4 ties). The Bayes factor for the associated sign test was 20,175. In the case of the parametric method, the ratio was 128 to 77, yielding a Bayes factor of 5611 (Table 1). While the Bayes factors for both implementations of the semi‐partial correlation were somewhat lower when the r.m.s. EMG values 50 and 10 ms prior to the stimuli were included as the covariate, in all cases they satisfied the Wetzels and Wagenmakers (2012) criteria to be deemed ‘strong evidence’ or ‘very strong evidence’.

Although the inclusion of ‘background EMG’ as a covariate reliably decreased the magnitude of the correlation coefficient, the adjusted values nonetheless suggested the presence of a positive monotonic association between the amplitudes of MEPs generated in rFCR and lFCR. Binomial expansions reflecting the probability of obtaining by chance alone the observed distributions of (covariate adjusted) positive and negative correlation coefficients were in all cases smaller than 2.10 × 10^−7^ (Table 1). Similarly, although the mean magnitudes of the rho values obtained through semi‐partial correlation were small (0.13–0.15), the corresponding confidence limits (Table 1) allow the presence of a positive association in the population to reasonably inferred.

In circumstances in which evoked potentials are polyphasic (as frequently observed for muscles in the forearm), it may be preferable in some cases to employ MEP area (rather than MEP amplitude) when seeking to quantify the short‐latency component of the response to cortical stimulation (Groppa et al., 2012). Measures of association were therefore also derived using the MEP area calculated for an epoch (12.5–45 ms following stimulation) appropriate to the duration of potentials evoked in FCR (Grospretre & Martin, 2014). For this purpose, the r.m.s. EMG recorded in each muscle for the 100 ms period in advance of stimulation was used. The outcomes were in very close accordance (see Table 1) with those obtained when the calculations were based on the (‘peak‐to‐peak’) amplitude of the MEPs. The unadjusted correlation coefficient, representing the association of the responses to the CS and the TS, was rho = 0.18 (95% CI = 0.14–0.22). The coefficient obtained when the parametric method was used to adjust for covariates was rho = 0.14 (95% CI = 0.10–0.17). When the non‐parametric method was used, the coefficient was rho = 0.14 (95% CI = 0.11–0.18).

The mean (across 205 participants) amplitude of the MEP generated by the TS alone was 102 µV. When preceded by the CS, the mean amplitude was 62 µV. The canonical finding of ‘inhibition’, that is, when estimated for each participant over series of responses, was therefore obtained.

The reader may also be interested in the magnitude of the association between the r.m.s. EMG recorded in each muscle (i.e. rFCR and lFCR) during the period in advance of the stimulation and the size of the evoked response. For the rFCR (i.e. in respect of the TS), the associations were: for the 100 ms period, rho = 0.15 (95% CI = 0.11–0.18); for the 50 ms period, rho = 0.13 (95% CI = 0.09–0.17); and for the 10 ms period, rho = 0.10 (95% CI = 0.07–0.14). For the lFCR (i.e. in respect of the CS), the associations were: for the 100 ms period, rho = 0.10 (95% CI = 0.07–0.14); for the 50 ms period, rho = 0.08 (95% CI = 0.04–0.12); and for the 10 ms period, rho = 0.05 (95% CI = 0.02–0.09).

## 4 DISCUSSION

### 4.1 The influence of variations in ‘background’ EMG

There is a key finding with significance beyond the context of the present study. It is that variations in ‘background’ EMG signals over a range that does not extend beyond an upper limit of 2µV have a systematic influence on the amplitude of MEPs. The magnitude of this influence is such that its removal alters substantially the magnitude of correlations between the amplitudes of MEPs recorded in homologous muscles of the upper limb, in response to near‐simultaneous TMS delivered over the motor cortices.

It is often the case that researchers reject trials if the r.m.s. EMG registered in a period (typically 50 or 100 ms) preceding the delivery of TMS exceeds a pre‐defined threshold. The thresholds employed in practice vary considerably. Although values of 10 µV (e.g. Mooney et al., 2025) and 20 µV (e.g. Vallence et al., 2023) are common, much higher thresholds of 50 µV (e.g. Castiglione & Aron, 2021; Duque & Ivry, 2009) or 100 µV (e.g. Duque et al., 2012, 2010) have also been utilized. The lowest known rejection threshold is that of 2.5 µV used by Calvert & Carson (2023). The assumption implicit in the adoption of these thresholds is that variations in the levels of signals recorded for retained trials reflect instrumentation or digitization noise (e.g. Li et al., 2022), rather than physiological sources. Specifically, for these trials the α‐motoneurones are treated as being silent. The present results suggest that this assumption may be ill‐founded, even if the rejection threshold is set as low as 2.5 µV.

Matthews (1999) demonstrated that for a single model motoneurone, an increase in background excitation from below firing threshold shifts the input–output function such that a relatively weak stimulus (a characterization that applies to the descending volley generated by most TMS protocols) is then more likely to induce a response. In relation to the motoneurone pools, the correlations observed in the present study – between pre‐stimulus r.m.s. EMG recorded from the left and right FCR – imply that a portion of the motoneurones innervating each muscle were in receipt of excitation sufficient to exceed firing thresholds and enter the ‘discharge zone’ (Lloyd, 1945). This state having been reached, the ‘excited zone’ (Lloyd, 1945) will also have included a ‘subliminal fringe’ – cells depolarized by the excitatory input, but not brought to firing threshold (Denny‐Brown & Sherrington, 1928). The size of the fringe is contingent on the net excitation of the pool. Descending input to motoneurone pools generated by cortical TMS will cause the discharge of action potentials (contributing to MEPs) in proportion to the size of the subliminal fringe (see also Theodosiadou et al., 2023). Changes in levels of excitation as small as those detected here, reflected in systematic (such as evidenced by correlations) trial‐to‐trial fluctuations in r.m.s. EMG over a range not exceeding 2 µV, will thus necessarily alter the amplitude of MEPs. Variations over greater ranges (e.g. extending to 20 or 100 µV) will have a commensurately larger impact.

### 4.2 Covariation in the excitability of corticospinal projections

A central aim of the present study was to determine whether the amplitudes of MEPs generated by successive stimulation of the two motor cortices are positively correlated. Following statistical compensation for the mediating influence on MEP amplitudes of fluctuations in the background EMG (remarked upon above), it was determined that positive correlations are observed predominantly (although not exclusively). Positive covariation of MEP amplitudes is in accordance with the hypothesis (Carson, 2020) that callosal fibres mediate the reciprocal shaping of excitation in homotopic brain centres with descending projections onto motoneurons that innervate homologous muscles. It is also consistent with reports, derived from magnetoencephalography (MEG) recordings that include ‘pure rest’ conditions, of synchronized activity in a highly focal complex that comprises bilateral primary motor cortex (M1) (O'Neill et al., 2015; see also Huang et al., 2014; Liu et al., 2010). The present findings suggest that bilateral synchronization can also be revealed by means of TMS. Some questions that remain include whether the magnitude of the covariation that can be detected is subject to, or reflective of, the influence of acute (e.g. task/fatigue related) or chronic (e.g. age/disease related) changes in state.

It is possible that the observed covariation reflects non‐specific physiological variations (such as level of arousal) that exert a moderating influence on corticospinal excitability. There is, for example, fragmentary evidence to suggest that the autonomic nervous system mediates an influence of cardiac activity on corticospinal excitability (Al et al., 2023; cf. Bianchini et al., 2021; Filippi et al., 2000). It would be anticipated that any such effects would be bilateral in their expression. Relatedly, it has been noted previously (Calvert & Carson, 2022) that elevations in the magnitude of the BOLD response reported for the opposite hemisphere during unilateral movement may arise as an artefact of correlated vasomotion.

Furthermore, the CS may exert an influence along the neuraxis via ipsilateral projections (e.g. corticoreticulospinal), such that the state of the spinal motoneuron pool (i.e. contralateral to the site of the TS) is altered prior to the arrival of the descending volley generated by the TS. To the extent that any such influence varies with the physiological efficacy of the CS, there may be a corresponding impact on covariation in the magnitudes of MEPs generated by the CS and TS. Thus, the specific physiological mechanisms that account for the observed bilateral associations of MEP amplitudes remain to be adequately elucidated.

### 4.3 Factors influencing the magnitudes of the correlation coefficients obtained

One of the objectives was to resolve with a high degree of statistical power the magnitude of any associations that may be present. Due to the large sample size (*n* = 205), the confidence intervals for the correlation coefficients were narrowly defined. Therefore, even though the central tendencies of the adjusted (for background EMG) rho values were small (0.13–0.15), it is possible to have a high degree of confidence that the effect (i.e. the positive association of MEP amplitudes) is present in the population. There are, however, several reasons to believe that these values underestimate the true magnitude of the effect.

Perhaps the most obvious consideration that the CS and the TS were not simultaneous but separated by 10 ms. Thus, to the extent that the observed associations reflect zero‐lag synchrony of interacting neural populations in the two hemispheres (Mehra et al., 2025; O'Reilly & Elsabbagh, 2021), the 10 ms interval may constitute a phase offset (i.e. between the CS and TS). Notwithstanding steps taken to systematize and stabilize the testing procedures, moment to moment changes in the position and angles (yaw, pitch, roll) of the coils with respect to the head, will have introduced variation in the biophysical efficacy of the CS and TS. To the extent that, on a given trial, any such changes did not affect equivalently the delivery of the CS and the TS, there will have been a negative impact on the registered correlation of MEP amplitudes.

It is likely to be especially significant that the CS and TS were differentiated with respect to both coil orientation and stimulation intensity. The TS was delivered at an intensity of 120% RMT using a coil orientation designed to induce P‐A current flow across the motor strip. In contrast, the CS was delivered at an intensity of 130% RMT, with the coil oriented to produce a current that flowed from lateral to medial (L‐M). It is well established that the direction of the induced current influences the manner in which magnetic stimulation excites corticospinal output cells (Mills et al., 1992; Sakai et al., 1997; Werhahn et al., 1994). The consensus (e.g. Siebner et al., 2022) is that magnetic stimuli which induce P‐A current flow predominantly evoke I‐ (or ‘indirect’) waves, including the I1‐element elicited by monosynaptic inputs to corticospinal neurons, whereas, at least when relatively high stimulation intensities are employed, L‐M induced current flow can evoke D‐waves. This term is used, as ‘direct’ excitation of the corticospinal axons has been inferred (Di Lazzaro & Rothwell, 2014). In practical application, such distinctions between coil orientations are of course relative (i.e. in terms of neuroanatomy) rather than absolute. The effective intensity of stimulation, that is, as it affects the brain, also has a significant bearing on the contribution of different classes of inhibitory and excitatory inputs to the corticospinal neurons (Di Lazzaro et al., 2012). The net effects of differences (between CS and TS) in coil orientation and stimulation intensity on the observed magnitudes of the correlation coefficients cannot be gauged directly. Taking all these factors into account, however, it might be supposed that the true extent of the (physiological) covariation may be greater than implied by the statistical measures of association reported here.

One can be confident that when the direction of induced current flow and the intensity of stimulation are different, the neuronal populations activated by dual hemisphere TMS, even when the coils are placed over matching scalp locations, will not be strictly homotopic. It is rather more difficult to characterize precisely the structural and functional identities of these populations. This is due, in part, to the poor spatial definition of TMS. Modelling based on motor potentials evoked by direct electrical stimulation (DES) of the exposed cortex during neurosurgery suggests that when TMS is delivered at 120% rMT, the stimulating effect spans an area of several cm^2^ and extends over one or two neighbouring gyri (Opitz et al., 2014). The current consensus is that not only neurons in the rostral M1 (at the precentral convexity of the central sulcus) but also in the caudal portion of the dorsal premotor cortex (PMd) are likely to be activated by TMS when the coil is placed at the site that is optimal for the elicitation of MEPs (Aberra et al., 2020; Dubbioso et al., 2021). Cortico‐cortical callosal projections from these zones terminate in both homotopic subregions of the opposite hemisphere (Innocenti et al., 2017) and in heterotopic regions (Chovsepian et al., 2017; Innocenti, 1986; Ruddy et al., 2017). In view of both this pattern of structural connectivity and the spatially distributed action of TMS, it would be presumptuous to suppose that (in an IHI paradigm) the transcallosal effects of the conditioning TMS are closely circumscribed. Soon it may become possible to advance our understanding by means of high‐density electroencephalography (EEG) systems capable of extremely high (e.g. 50 kHz, Beck et al., 2024) sampling rates sufficient to curtail the duration of the artefact generated by TMS and permit resolution of the distributed effects of the stimulation on neuronal populations in the opposite hemisphere (e.g. Guidali et al., 2023; Lucarelli et al., 2025).

### 4.4 Future directions

As noted above, the positive correlations between the amplitudes of MEPs recorded in the left and right FCR are consistent with observations (including those derived from intracranial recordings) of minimal lag synchronized activity in homotopic brain regions (O'Reilly & Elsabbagh, 2021). In this vein, Stefanou et al. (2018) derived a measure of IHI from groups of trials that were classified in terms of the μ‐rhythms (typical range 7.5–12.5 Hz) registered via scalp electrode montages centred over left and right M1. It was noted that the largest IHI estimates were seen for groups of trials on which the two μ‐rhythms (left and right) were in‐phase, and for which both the CS and TS were delivered close to the negative peak of the respective (i.e. hemisphere specific) cycles. There is, however, a further consideration. It is that the CS induces synchronization within a network that extends to the opposite M1 (Momi et al., 2022), and by this means alters the transient state of neural populations that influence (subsequent) TS excitation of corticospinal output cells. Unfortunately, due to features inherent to most EEG recording systems that restrict the period following the delivery of TMS during which recordings of brain activity can be resolved, there are (to the best of my knowledge) no published data that pertain to stimulation‐induced inter‐hemispheric synchronization in human within an IHI‐typical 10 ms interval, which would allow this conjecture to be assessed (cf. Guidali et al., 2023; Lucarelli et al., 2025). In the event that the positive covariation of MEP amplitudes reported here is contingent on phase resetting (Nuyts et al., 2025; Winfree, 1980) (i.e. of neural activity in the opposite hemisphere) induced by the CS, it might be concluded that the phenomenon described is an artefact of TMS induced neural excitation, rather than a reflection of normal physiological processes. A protocol in which simultaneous stimuli are used therefore has the potential to exclude this possibility. This is a possible direction for future research. A simultaneous stimulation protocol is also likely to confer advantages (should the association remain present) in evaluating the hypothesis that its mediation is via minimal lag synchronized activity in homotopic brain regions, particularly when combined with new methodologies that permit TMS‐evoked EEG activity to be registered at very short latencies (e.g. Beck et al., 2024).

### 4.5 Conclusions

There is positive covariation in the amplitudes of MEPs generated by (near simultaneous) stimulation of the two motor cortices. Some of this covariation arises from correlated bilateral fluctuations in the excitability of spinal motoneurones. It nonetheless remains consistently expressed following statistical compensation for these fluctuations. The presence of positive covariation does not accord with the conjecture that each cerebral hemisphere has a suppressing influence upon its opposite counterpart. It is, however, consistent with the presumed presence of highly focal synchronized bilateral activity in a neural ensemble that encompasses the two primary motor cortices (O'Neill et al., 2015). An alternative possibility is that the positive covariation of MEP amplitudes obtained using an IHI paradigm arises from phase resetting by the CS of neural populations that influence the subsequent TS excitation of corticospinal output cells.

As a general point, it should also be acknowledged that measures derived from many electrophysiological assays applied when the participant is ‘at rest’ are not necessarily indicative of the physiological processes that are operative in the context of natural movements (Calvert & Carson, 2022; Carson et al., 2016). This point applies with especial force to the electrophysiological phenomenon of ‘inter‐hemispheric inhibition’ (Carson, 2020).

## AUTHOR CONTRIBUTIONS

Sole author.

## CONFLICT OF INTEREST

None declared.

## FUNDING INFORMATION

None.

## Data Availability

Example R code and data, which can be used to reproduce the methods of analysis described herein, is available via: https://zenodo.org/doi/10.5281/zenodo.17397343. Due to the nature of the consent given by the individuals who participated in this study, it is not possible to make the individual recordings publicly available.

## References

[eph70098-bib-0001] Aberra, A. S. , Wang, B. , Grill, W. M. , & Peterchev, A. V. (2020). Simulation of transcranial magnetic stimulation in head model with morphologically‐realistic cortical neurons. Brain Stimulation, 13(1), 175–189. 31611014 10.1016/j.brs.2019.10.002 PMC6889021

[eph70098-bib-0002] Al, E. , Stephani, T. , Engelhardt, M. , Haegens, S. , Villringer, A. , & Nikulin, V. V. (2023). Cardiac activity impacts cortical motor excitability. PLoS Biology, 21(11), e3002393. 38015826 10.1371/journal.pbio.3002393 PMC10684011

[eph70098-bib-0003] Asanuma, H. , & Okamoto, K. (1959). Unitary study on evoked activity of callosal neurons and its effect on pyramidal tract cell activity on cats. The Japanese Journal of Physiology, 9(4), 473–483. 13794638 10.2170/jjphysiol.9.473

[eph70098-bib-0004] Awiszus, F. (2003). TMS and threshold hunting. In Supplements to clinical neurophysiology, (Vol. 56, pp. 13–23). Elsevier. 14677378 10.1016/s1567-424x(09)70205-3

[eph70098-bib-0005] Awiszus, F. , & Borckardt, J. (2011). TMS motor threshold assessment tool (MTAT 2.0). http://www.clinicalresearcher.org/software.htm

[eph70098-bib-0006] Barker, A. T. , Jalinous, R. , & Freeston, I. L. (1985). Non‐invasive magnetic stimulation of human motor cortex. The Lancet, 325(8437), 1106–1107. 10.1016/s0140-6736(85)92413-4 2860322

[eph70098-bib-0007] Bäumer, T. , Bock, F. , Koch, G. , Lange, R. , Rothwell, J. C. , Siebner, H. R. , & Münchau, A. (2006). Magnetic stimulation of human premotor or motor cortex produces interhemispheric facilitation through distinct pathways. The Journal of Physiology, 572(3), 857–868. 16497712 10.1113/jphysiol.2006.104901 PMC1780006

[eph70098-bib-0008] Beck, M. M. , Christiansen, L. , Madsen, M. A. J. , Jadidi, A. F. , Vinding, M. C. , Thielscher, A. , Bergmann, T. O. , Siebner, H. R. , & Tomasevic, L. (2024). Transcranial magnetic stimulation of primary motor cortex elicits an immediate transcranial evoked potential. Brain Stimulation, 17(4), 802–812. 38909748 10.1016/j.brs.2024.06.008

[eph70098-bib-0009] Belyk, M. , Banks, R. , Tendera, A. , Chen, R. , & Beal, D. S. (2021). Paradoxical facilitation alongside interhemispheric inhibition. Experimental Brain Research, 239(11), 3303–3313. 34476535 10.1007/s00221-021-06183-9 PMC8541949

[eph70098-bib-0010] Bianchini, E. , Mancuso, M. , Zampogna, A. , Guerra, A. , & Suppa, A. (2021). Cardiac cycle does not affect motor evoked potential variability: A real‐time EKG‐EMG study. Brain Stimulation, 14(1), 170–172. 33359602 10.1016/j.brs.2020.12.009

[eph70098-bib-0011] Bianki, V. L. (1981). Interaction between transcallosal and thalamocortical excitation. Neuroscience and Behavioral Physiology, 11(4), 328–335. 6283417 10.1007/BF01184194

[eph70098-bib-0012] Boonstra, T. W. , Daffertshofer, A. , Van Ditshuizen, J. C. , Van den Heuvel, M. R. C. , Hofman, C. , Willigenburg, N. W. , & Beek, P. J. (2008). Fatigue‐related changes in motor‐unit synchronization of quadriceps muscles within and across legs. Journal of Electromyography and Kinesiology, 18(5), 717–731. 17462912 10.1016/j.jelekin.2007.03.005

[eph70098-bib-0013] Calvert, G. H. , & Carson, R. G. (2022). Neural mechanisms mediating cross education: With additional considerations for the ageing brain. Neuroscience & Biobehavioral Reviews, 132, 260–288. 34801578 10.1016/j.neubiorev.2021.11.025

[eph70098-bib-0014] Calvert, G. H. , & Carson, R. G. (2023). Induction of interhemispheric facilitation by short bursts of transcranial alternating current stimulation. Neuroscience Letters, 803, 137190. 36921664 10.1016/j.neulet.2023.137190

[eph70098-bib-0015] Carson, R. G (2020). Inter‐hemispheric inhibition sculpts the output of neural circuits by co‐opting the two cerebral hemispheres. The Journal of Physiology, 598(21), 4781–4802. 32770748 10.1113/JP279793

[eph70098-bib-0016] Carson, R. G. , Ruddy, K. L. , & McNickle, E. (2016). What do TMS‐evoked motor potentials tell us about motor learning? Advances in Experimental Medicine and Biology, 957, 143–157. 28035564 10.1007/978-3-319-47313-0_8

[eph70098-bib-0017] Castiglione, A. , & Aron, A. R. (2021). Unwanted memory intrusions recruit broad motor suppression. Journal of Cognitive Neuroscience, 33(1), 119–128. 33078991 10.1162/jocn_a_01642 PMC9054000

[eph70098-bib-0018] Chechile, R. A. (2020). Bayesian Statistics for Experimental Scientists: A General Introduction Using Distribution_Free Methods. MIT Press.

[eph70098-bib-0019] Chiarello, C. , & Maxfield, L. (1996). Varieties of interhemispheric inhibition, or how to keep a good hemisphere down. Brain and cognition, 30(1), 81–108. 8811982 10.1006/brcg.1996.0006

[eph70098-bib-0020] Chovsepian, A. , Empl, L. , Correa, D. , & Bareyre, F. M. (2017). Heterotopic transcallosal projections are present throughout the mouse cortex. Frontiers in Cellular Neuroscience, 11, 36. 28270750 10.3389/fncel.2017.00036 PMC5318386

[eph70098-bib-0021] Denny‐Brown, D. E. , & Sherrington, C. S. (1928). Subliminal fringe in spinal flexion. The Journal of Physiology, 66(2), 175–180. 16993981 10.1113/jphysiol.1928.sp002516 PMC1402734

[eph70098-bib-0022] Di Lazzaro, V. , Profice, P. , Ranieri, F. , Capone, F. , Dileone, M. , Oliviero, A. , & Pilato, F. (2012). I‐wave origin and modulation. Brain Stimulation, 5(4), 512–525. 21962980 10.1016/j.brs.2011.07.008

[eph70098-bib-0023] Di Lazzaro, V. , & Rothwell, J. C. (2014). Corticospinal activity evoked and modulated by non‐invasive stimulation of the intact human motor cortex. The Journal of Physiology, 592(19), 4115–4128. 25172954 10.1113/jphysiol.2014.274316 PMC4215763

[eph70098-bib-0024] Dubbioso, R. , Madsen, K. H. , Thielscher, A. , & Siebner, H. R. (2021). The Myelin Content of the Human Precentral Hand Knob Reflects Interindividual Differences in Manual Motor Control at the Physiological and Behavioral Level. Journal of Neuroscience, 41(14), 3163–3179. 33653698 10.1523/JNEUROSCI.0390-20.2021 PMC8026359

[eph70098-bib-0025] Duque, J. , & Ivry, R. B. (2009). Role of corticospinal suppression during motor preparation. Cerebral Cortex, 19(9), 2013–2024. 19126798 10.1093/cercor/bhn230 PMC2722425

[eph70098-bib-0026] Duque, J. , Labruna, L. , Verset, S. , Olivier, E. , & Ivry, R. B. (2012). Dissociating the Role of Prefrontal and Premotor Cortices in Controlling Inhibitory Mechanisms during Motor Preparation. The Journal of Neuroscience, 32(3), 806–816. 22262879 10.1523/JNEUROSCI.4299-12.2012 PMC3304578

[eph70098-bib-0027] Duque, J. , Lew, D. , Mazzocchio, R. , Olivier, E. , & Ivry, R. B. (2010). Evidence for two concurrent inhibitory mechanisms during response preparation. The Journal of Neuroscience, 30(10), 3793–3802. 20220014 10.1523/JNEUROSCI.5722-09.2010 PMC2852647

[eph70098-bib-0028] Duque, J. , Murase, N. , Celnik, P. , Hummel, F. , Harris‐Love, M. , Mazzocchio, R. , Olivier, E. , & Cohen, L. G. (2007). Intermanual differences in movement‐related interhemispheric inhibition. Journal of Cognitive Neuroscience, 19(2), 204–213. 17280510 10.1162/jocn.2007.19.2.204

[eph70098-bib-0029] Ferbert, A. , Priori, A. , Rothwell, J. C. , Day, B. L. , Colebatch, J. G. , & Marsden, C. D. (1992). Interhemispheric inhibition of the human motor cortex. The Journal of Physiology, 453(1), 525–546. 1464843 10.1113/jphysiol.1992.sp019243 PMC1175572

[eph70098-bib-0030] Ferguson, G. A (1971). Statistical analysis in psychology and education. McGraw‐Hill.

[eph70098-bib-0031] Filippi, M. M. , Oliveri, M. , Vernieri, F. , Pasqualetti, P. , & Rossini, P. M. (2000). Are autonomic signals influencing cortico‐spinal motor excitability?: A study with transcranial magnetic stimulation. Brain Research, 881(2), 159–164. 11036154 10.1016/s0006-8993(00)02837-7

[eph70098-bib-0032] Groppa, S. , Oliviero, A. , Eisen, A. , Quartarone, A. , Cohen, L. G. , Mall, V. , Kaelin‐Lang, A. , Mima, T. , Rossi, S. , Thickbroom, G. W. , Rossini, P. M. , Ziemann, U. , Valls‐Solé, J. , & Siebner, H. R. (2012). A practical guide to diagnostic transcranial magnetic stimulation: Report of an IFCN committee. Clinical Neurophysiology, 123(5), 858–882. 22349304 10.1016/j.clinph.2012.01.010 PMC4890546

[eph70098-bib-0033] Grospretre, S. , & Martin, A. (2014). Conditioning effect of transcranial magnetic stimulation evoking motor‐evoked potential on V‐wave response. Physiological Reports, 2(12), e12191. 25501438 10.14814/phy2.12191 PMC4332197

[eph70098-bib-0034] Guidali, G. , Zazio, A. , Lucarelli, D. , Marcantoni, E. , Stango, A. , Barchiesi, G. , & Bortoletto, M. (2023). Effects of transcranial magnetic stimulation (TMS) current direction and pulse waveform on cortico‐cortical connectivity: A registered report TMS‐EEG study. European Journal of Neuroscience, 58(8), 3785–3809. 37649453 10.1111/ejn.16127

[eph70098-bib-0035] Hanajima, R. , Ugawa, Y. , Machii, K. , Mochizuki, H. , Terao, Y. , Enomoto, H. , Furubayashi, T. , Shiio, Y. , Uesugi, H. , & Kanazawa, I. (2001). Interhemispheric facilitation of the hand motor area in humans. The Journal of Physiology, 531(3), 849–859. 11251064 10.1111/j.1469-7793.2001.0849h.x PMC2278503

[eph70098-bib-0036] Harrell, F. E. (2015). Regression modeling strategies: With applications to linear models, logistic regression, and survival analysis, (Vol. 608). 2nd ed. Springer.

[eph70098-bib-0037] Hellige, J. B. (1993). Hemispheric asymmetry: What’ s right and what's left. Harvard University Press.

[eph70098-bib-0038] Hess, C. W. , Mills, K. R. , & Murray, N. M. (1987). Responses in small hand muscles from magnetic stimulation of the human brain. The Journal of Physiology, 388(1), 397–419. 3079553 10.1113/jphysiol.1987.sp016621 PMC1192555

[eph70098-bib-0039] Huang, M. X. , Huang, C. W. , Robb, A. , Angeles, A. , Nichols, S. L. , Baker, D. G. , Song, T. , Harrington, D. L. , Theilmann, R. J. , Srinivasan, R. , Heister, D. , Diwakar, M. , Canive, J. M. , Edgar, J. C. , Chen, Y. H. , Ji, Z. , Shen, M. , El‐Gabalawy, F. , Levy, M. , … Lee, R. R. (2014). MEG source imaging method using fast L1 minimum‐norm and its applications to signals with brain noise and human resting‐state source amplitude images. Neuroimage, 84, 585–604. 24055704 10.1016/j.neuroimage.2013.09.022 PMC4096863

[eph70098-bib-0040] Ibey, R. J. , Bolton, D. A. , Buick, A. R. , Staines, W. R. , & Carson, R. G. (2015). Interhemispheric inhibition of corticospinal projections to forearm muscles. Clinical Neurophysiology, 126(10), 1934–1940. 25561164 10.1016/j.clinph.2014.12.006

[eph70098-bib-0041] Innocenti, G. M. (1986). General organization of callosal connections in the cerebral cortex. In E. G. Jones , & A. Peters , (Eds.). Cerebral cortex, (pp. 291–353). Plenum Press.

[eph70098-bib-0042] Innocenti, G. M. , Dyrby, T. B. , Andersen, K. W. , Rouiller, E. M. , & Caminiti, R. (2017). The crossed projection to the striatum in two species of monkey and in humans: Behavioral and evolutionary significance. Cerebral Cortex, 27(6), 3217–3230. 27282154 10.1093/cercor/bhw161

[eph70098-bib-0043] Kammer, T. , Beck, S. , Thielscher, A. , Laubis‐Herrmann, U. , & Topka, H. (2001). Motor thresholds in humans: A transcranial magnetic stimulation study comparing different pulse waveforms, current directions and stimulator types. Clinical Neurophysiology, 112(2), 250–258. 11165526 10.1016/s1388-2457(00)00513-7

[eph70098-bib-0044] Karacan, I. , Arslan, B. T. , Karaoglu, A. , Aydin, T. , Gray, S. , Ungan, P. , & Türker, K. S. (2023). Estimating and minimizing movement artifacts in surface electromyogram. Journal of Electromyography and Kinesiology, 70, 102778. 37141730 10.1016/j.jelekin.2023.102778

[eph70098-bib-0045] Kavanagh, J. J. , Cresswell, A. G. , Sabapathy, S. , & Carroll, T. J. (2013). Bilateral tremor responses to unilateral loading and fatiguing muscle contractions. Journal of Neurophysiology, 110(2), 431–440. 23636728 10.1152/jn.00228.2013

[eph70098-bib-0046] Kinsbourne, M. (1974). Lateral interactions in the brain. In M. Kinsbourne , & W. L. Smith , (Eds.), Hemispheric disconnection and cerebral function, (pp. 239–259). Charles C Thomas.

[eph70098-bib-0047] Li, C. , & Shepherd, B. E. (2012). A new residual for ordinal outcomes. Biometrika, 99(2), 473–480. 23843667 10.1093/biomet/asr073 PMC3635659

[eph70098-bib-0048] Li, Z. , Peterchev, A. V. , Rothwell, J. C. , & Goetz, S. M. (2022). Detection of motor‐evoked potentials below the noise floor: Rethinking the motor stimulation threshold. Journal of Neural Engineering, 19(5), 056040. 10.1088/1741-2552/ac7dfc PMC10155352 35785762

[eph70098-bib-0049] Limbert, E. , Stahel, W. A. , & Abbt, M. (2001). Log‐normal Distributions across the Sciences: Keys and Clues. Bioscience, 51(5), 341–352.

[eph70098-bib-0050] Liu, Q. , Li, C. , Wanga, V. , & Shepherd, B. E. (2018). Covariate‐adjusted Spearman's rank correlation with probability‐scale residuals. Biometrics, 74(2), 595–605. 29131931 10.1111/biom.12812 PMC5949238

[eph70098-bib-0051] Liu, Q. , Shepherd, B. , & Li, C. (2020). PResiduals: An R package for residual analysis using probability‐scale residuals. Journal of Statistical Software, 94(12), 1–27. 10.18637/jss.v094.i12 PMC11451338 39371079

[eph70098-bib-0052] Liu, Z. , Fukunaga, M. , de Zwart, J. A. , & Duyn, J. H. (2010). Large‐scale spontaneous fluctuations and correlations in brain electrical activity observed with magnetoencephalography. Neuroimage, 51(1), 102–111. 20123024 10.1016/j.neuroimage.2010.01.092 PMC2847019

[eph70098-bib-0053] Lloyd, D. P. (1945). On the relation between discharge zone and subliminal fringe in a motoneuron pool supplied by a homogeneous presynaptic pathway. The Yale Journal of Biology and Medicine, 18(2), 117–121. 21007545 PMC2601793

[eph70098-bib-0054] Lucarelli, D. , Guidali, G. , Sulcova, D. , Zazio, A. , Bonfiglio, N. S. , Stango, A. , Barchiesi, G. , & Bortoletto, M. (2025). Stimulation parameters recruit distinct cortico‐cortical pathways: Insights from microstate analysis on tms‐evoked potentials. Brain Topography, 38(3), 39. 40153104 10.1007/s10548-025-01113-2 PMC11953218

[eph70098-bib-0055] Maertens de Noordhout, A. , Pepin, J. L. , Gerard, P. , & Delwaide, P. J. (1992). Facilitation of responses to motor cortex stimulation: Effects of isometric voluntary contraction. Annals of Neurology, 32(3), 365–370. 1416805 10.1002/ana.410320310

[eph70098-bib-0056] Matthews, P. B. C. (1999). The effect of firing on the excitability of a model motoneurone and its implications for cortical stimulation. The Journal of Physiology, 518(3), 867–882. 10420021 10.1111/j.1469-7793.1999.0867p.x PMC2269455

[eph70098-bib-0057] Mehra, C. , Beyh, A. , Laiou, P. , Garces, P. , Jones, E. J. , Mason, L. , Buitelaar, J. , Johnson, M. H. , Murphy, D. , Loth, E. , Dell'Acqua, F. , Ewen, J. B. , Richardson, M. P. , & O'Muircheartaigh, J. (2025). Zero‐phase‐delay synchrony between interacting neural populations: Implications for functional connectivity derived biomarkers. BioRxiv. 10.1101/2025.01.04.631256 PMC12603661 41230416

[eph70098-bib-0058] Mills, K. R. , Boniface, S. J. , & Schubert, M. (1992). Magnetic brain stimulation with a double coil: The importance of coil orientation. Electroencephalography and Clinical Neurophysiology/Evoked Potentials Section, 85(1), 17–21. 10.1016/0168-5597(92)90096-t 1371739

[eph70098-bib-0059] Momi, D. , Ozdemir, R. A. , Tadayon, E. , Boucher, P. , Di Domenico, A. , Fasolo, M. , Shafi, M. M. , Pascual‐Leone, A. , & Santarnecchi, E. (2022). Phase‐dependent local brain states determine the impact of image‐guided transcranial magnetic stimulation on motor network electroencephalographic synchronization. The Journal of Physiology, 600(6), 1455–1471. 34799873 10.1113/JP282393 PMC9728936

[eph70098-bib-0060] Mooney, R. A. , Anaya, M. A. , Stilling, J. M. , & Celnik, P. A. (2025). Heightened Reticulospinal Excitability after Severe Corticospinal Damage in Chronic Stroke. Annals of Neurology, 97(1), 163–174. 10.1002/ana.27103 39387284

[eph70098-bib-0061] Moscovitch, M. (1976). On the representation of language in the right hemisphere of right‐handed people. Brain and Language, 3(1), 47–71. 1268698 10.1016/0093-934x(76)90006-7

[eph70098-bib-0062] Nielsen, J. F. (1996). Logarithmic distribution of amplitudes of compound muscle action potentials evoked by transcranial magnetic stimulation. Journal of Clinical Neurophysiology, 13(5), 423–434. 8897207 10.1097/00004691-199609000-00005

[eph70098-bib-0063] Nuyts, M. , Beck, M. M. , Banach, A. , Thielscher, A. , Meesen, R. , Tomasevic, L. , Siebner, H. R. , & Christiansen, L. (2025). Rostro‐caudal TMS mapping of immediate transcranial evoked potentials reveals a pericentral crescendo‐decrescendo pattern. Neuroimage, 319, 121446. 40921260 10.1016/j.neuroimage.2025.121446

[eph70098-bib-0064] Oldfield, R. C. (1971). The assessment and analysis of handedness: The Edinburgh inventory. Neuropsychologia, 9(1), 97–113. 5146491 10.1016/0028-3932(71)90067-4

[eph70098-bib-0065] O'Neill, G. C. , Bauer, M. , Woolrich, M. W. , Morris, P. G. , Barnes, G. R. , & Brookes, M. J. (2015). Dynamic recruitment of resting state sub‐networks. Neuroimage, 115, 85–95. 25899137 10.1016/j.neuroimage.2015.04.030 PMC4573462

[eph70098-bib-0066] Opitz, A. , Zafar, N. , Bockermann, V. , Rohde, V. , & Paulus, W. (2014). Validating computationally predicted TMS stimulation areas using direct electrical stimulation in patients with brain tumors near precentral regions. NeuroImage: Clinical, 4, 500–507. 24818076 10.1016/j.nicl.2014.03.004 PMC3984442

[eph70098-bib-0067] O'Reilly, C. , & Elsabbagh, M. (2021). Intracranial recordings reveal ubiquitous in‐phase and in‐antiphase functional connectivity between homotopic brain regions in humans. Journal of Neuroscience Research, 99(3), 887–897. 33190333 10.1002/jnr.24748

[eph70098-bib-0068] Perotto, A. O. (1994). Anatomical guide for the electromyographer: The limbs and trunk. 3rd ed. Charles C. Thomas.

[eph70098-bib-0069] R Core Team . (2023). R: A language and environment for statistical computing. R Foundation for Statistical Computing. https://www.R‐project.org/

[eph70098-bib-0070] Rossi, S. , Antal, A. , Bestmann, S. , Bikson, M. , Brewer, C. , Brockmöller, J. , Carpenter, L. L. , Cincotta, M. , Chen, R. , Daskalakis, J. D. , Di Lazzaro, V. , Fox , M. D. , George, M. S. , Gilbert, D. , Kimiskidis, V. K. , Koch, G. , Ilmoniemi, R. J. , Lefaucheur, J. P. , Leocani, L. , … Hallett, M. (2021). Safety and recommendations for TMS use in healthy subjects and patient populations, with updates on training, ethical and regulatory issues: Expert Guidelines. Clinical Neurophysiology, 132(1), 269–306. 33243615 10.1016/j.clinph.2020.10.003 PMC9094636

[eph70098-bib-0071] Rossi, S. , Hallett, M. , Rossini, P. M. , Pascual‐Leone, A. , & Safety of TMS Consensus Group . (2009). Safety, ethical considerations, and application guidelines for the use of transcranial magnetic stimulation in clinical practice and research. Clinical Neurophysiology, 120(12), 2008–2039. 19833552 10.1016/j.clinph.2009.08.016 PMC3260536

[eph70098-bib-0072] Rothwell, J. C. , Thompson, P. D. , Day, B. L. , Boyd, S. , & Marsden, C. D. (1991). Stimulation of the human motor cortex through the scalp. Experimental Physiology, 76(2), 159–200. 2059424 10.1113/expphysiol.1991.sp003485

[eph70098-bib-0073] Rothwell, J. C. , Thompson, P. D. , Day, B. L. , Dick, J. P. , Kachi, T. , Cowan, J. M. , & Marsden, C. D. (1987). Motor cortex stimulation in intact man. 1. General characteristics of EMG responses in different muscles. Brain, 110(5), 1173–1190. 3676697 10.1093/brain/110.5.1173

[eph70098-bib-0074] Ruddy, K. L. , Leemans, A. , & Carson, R. G. (2017). Transcallosal connectivity of the human cortical motor network. Brain Structure and Function, 222(3), 1243–1252. 27469272 10.1007/s00429-016-1274-1 PMC5368198

[eph70098-bib-0075] Ruddy, K. L. , Rudolf, A. K. , Kalkman, B. , King, M. , Daffertshofer, A. , Carroll, T. J. , & Carson, R. G. (2016). Neural adaptations associated with interlimb transfer in a ballistic wrist flexion task. Frontiers in Human Neuroscience, 10, 204. 27199722 10.3389/fnhum.2016.00204 PMC4853797

[eph70098-bib-0076] Rupasov, V. I. , Lebedev, M. A. , Erlichman, J. S. , & Linderman, M. (2012). Neuronal variability during handwriting: Lognormal distribution. PLoS ONE, 7(4), e34759. 22514664 10.1371/journal.pone.0034759 PMC3326033

[eph70098-bib-0077] Sakai, K. , Ugawa, Y. , Terao, Y. , Hanajima, R. , Furubayashi, T. , & Kanazawa, I. (1997). Preferential activation of different I waves by transcranial magnetic stimulation with a figure‐of‐eight‐shaped coil. Experimental Brain Research, 113(1), 24–32. 9028772 10.1007/BF02454139

[eph70098-bib-0078] Sebik, O. , Karacan, I. , Cidem, M. , & Türker, K. S. (2013). Rectification of SEMG as a tool to demonstrate synchronous motor unit activity during vibration. Journal of Electromyography and Kinesiology, 23(2), 275–284. 23098913 10.1016/j.jelekin.2012.09.009

[eph70098-bib-0079] Shepherd, B. E. , Li, C. , & Liu, Q. (2016). Probability‐scale residuals for continuous, discrete, and censored data. Canadian Journal of Statistics, 44(4), 463–479. 10.1002/cjs.11302 PMC5364820 28348453

[eph70098-bib-0080] Siebner, H. R. , Funke, K. , Aberra, A. S. , Antal, A. , Bestmann, S. , Chen, R. , Classen, J. , Davare, M. , Di Lazzaro, V. , Fox, P. T. , Hallett, M. , Karabanov, A. N. , Kesselheim, J. , Beck, M. M. , Koch, G. , Liebetanz, D. , Meunier, S. , Miniussi, C. , Paulus, W. , … Ugawa, Y. (2022). Transcranial magnetic stimulation of the brain: What is stimulated?–A consensus and critical position paper. Clinical Neurophysiology, 140, 59–97. 35738037 10.1016/j.clinph.2022.04.022 PMC9753778

[eph70098-bib-0081] Stefanou, M. I. , Desideri, D. , Belardinelli, P. , Zrenner, C. , & Ziemann, U. (2018). Phase synchronicity of μ‐rhythm determines efficacy of interhemispheric communication between human motor cortices. Journal of Neuroscience, 38(49), 10525–10534. 30355634 10.1523/JNEUROSCI.1470-18.2018 PMC6596251

[eph70098-bib-0082] Theodosiadou, A. , Henry, M. , Duchateau, J. , & Baudry, S. (2023). Revisiting the use of Hoffmann reflex in motor control research on humans. European Journal of Applied Physiology, 123(4), 695–710. 36571622 10.1007/s00421-022-05119-7

[eph70098-bib-0083] Ugawa, Y. , Hanajima, R. , & Kanazawa, I. (1993). Interhemispheric facilitation of the hand area of the human motor cortex. Neuroscience letters, 160(2), 153–155. 8247346 10.1016/0304-3940(93)90401-6

[eph70098-bib-0084] Vallence, A. M. , Rurak, B. K. , Fujiyama, H. , & Hammond, G. R. (2023). Covariation of the amplitude and latency of motor evoked potentials elicited by transcranial magnetic stimulation in a resting hand muscle. Experimental Brain Research, 241(3), 927–936. 36811686 10.1007/s00221-023-06575-z PMC9985579

[eph70098-bib-0085] Vercauteren, K. , Pleysier, T. , Van Belle, L. , Swinnen, S. P. , & Wenderoth, N. (2008). Unimanual muscle activation increases interhemispheric inhibition from the active to the resting hemisphere. Neuroscience Letters, 445(3), 209–213. 18793696 10.1016/j.neulet.2008.09.013

[eph70098-bib-0086] Werhahn, K. J. , Fong, J. K. Y. , Meyer, B. U. , Priori, A. , Rothwell, J. C. , Day, B. L. , & Thompson, P. D. (1994). The effect of magnetic coil orientation on the latency of surface EMG and single motor unit responses in the first dorsal interosseous muscle. Electroencephalography and Clinical Neurophysiology/Evoked Potentials Section, 93(2), 138–146. 10.1016/0168-5597(94)90077-9 7512920

[eph70098-bib-0087] Wetzels, R. , & Wagenmakers, E.‐J. (2012). A default Bayesian hypothesis test for correlations and partial correlations. Psychonomic Bulletin and Review, 19(6), 1057–1064. 22798023 10.3758/s13423-012-0295-x PMC3505519

[eph70098-bib-0088] Winfree, A. T. (1980). The Geometry of Biological Time. Springer.

[eph70098-bib-0089] Zult, T. , Goodall, S. , Thomas, K. , Hortobágyi, T. , & Howatson, G. (2015). Mirror illusion reduces motor cortical inhibition in the ipsilateral primary motor cortex during forceful unilateral muscle contractions. Journal of Neurophysiology, 113(7), 2262–2270. 25632077 10.1152/jn.00686.2014 PMC4416555

